# Propensity Score Analysis Assessing the Burden of Non-Communicable Diseases among the Transgender Population in the United States Using the Behavioral Risk Factor Surveillance System (2017–2019)

**DOI:** 10.3390/healthcare9060696

**Published:** 2021-06-09

**Authors:** Jennifer R. Pharr, Kavita Batra

**Affiliations:** 1 Department of Environmental and Occupational Health, School of Public Health, University of Nevada, Las Vegas, NV 89119, USA; 2 Office of Research, Kirk Kerkorian School of Medicine, University of Nevada, Las Vegas, NV 89102, USA; Kavita.batra@unlv.edu

**Keywords:** non-communicable diseases, transgender, propensity score matching, Behavioral Risk Factor Surveillance System

## Abstract

Research to assess the burden of non-communicable diseases (NCDs) among the transgender population needs to be prioritized given the high prevalence of chronic conditions and associated risk factors in this group. Previous cross-sectional studies utilized unmatched samples with a significant covariate imbalance resulting in a selection bias. Therefore, this cross-sectional study attempts to assess and compare the burden of NCDs among propensity score-matched transgender and cisgender population groups. This study analyzed Behavioral Risk Factor Surveillance System data (2017–2019) using complex weighting procedures to generate nationally representative samples. Logistic regression was fit to estimate propensity scores. Transgender and cisgender groups were matched by sociodemographic variables using a 1:1 nearest neighbor matching algorithm. McNemar, univariate, and multivariate logistic regression analyses were conducted among matched cohorts using R and SPSS version 26 software. Compared with the cisgender group, the transgender group was significantly more likely to have hypertension (31.3% vs. 27.6%), hypercholesteremia (30.8% vs. 23.7%), prediabetes (17.3% vs. 10.3%), and were heavy drinkers (6.7% vs. 6.0%) and smokers (22.4% vs. 20.0%). Moreover, the transgender group was more than twice as likely to have depression (aOR: 2.70, 95% CI 2.62–2.72), stroke (aOR: 2.52 95% CI 2.50–2.55), coronary heart disease (aOR: 2.77, 95% CI 2.74–2.81), and heart attack (aOR: 2.90, 95% CI 2.87–2.94). Additionally, the transgender group was 1.2–1.7 times more likely to have metabolic and malignant disorders. Differences were also found between transgender subgroups compared with the cisgender group. This study provides a clear picture of the NCD burden among the transgender population. These findings offer an evidence base to build health equity models to reduce disparities among transgender groups.

## 1. Introduction

In 2019, seven of the top ten leading causes of death worldwide were non-communicable diseases (NCDs) [1,2]. These include heart disease, stroke, chronic obstructive pulmonary disease, lung cancers, dementia, diabetes, and kidney diseases [3]. NCDs combined accounted for nearly three-quarters of all deaths globally the same year. Of the 40 million deaths due to NCDs, approximately 17 million deaths (42.5%) occur in individuals below 70 years of age and are considered premature [4]. Because of the overwhelming burden of NCD-associated mortality and the loss of life prematurely, Sustainable Development Goal 3.4 is to reduce death from NCDs by one-third through prevention and early detection by 2030 [5]. The majority of NCD-associated deaths are preventable and can be reduced by controlling modifiable risk factors, including smoking tobacco, alcohol consumption, high salt intake, obesity, hypertension, and hyperglycemia. According to previous prediction modeling, 37 million deaths could be prevented or delayed in a 15-year time frame by prioritizing the reduction in risk factors through a coordinated approach [6].

While the rising incidence of NCDs is a global issue, these also impact death, disability, and the economy in the United States (U.S.). NCDs dominate the top ten leading causes of death in the U.S., and include heart disease, cancer, chronic lower respiratory diseases (chronic bronchitis, emphysema, and asthma), stroke, Alzheimer’s disease, diabetes, and kidney disease [7]. Heart disease and stroke alone account for one-third of all deaths and result in an estimated $214 billion in healthcare cost and $138 billion in lost productivity [8,9]. An additional 600,000 American lives are lost each year to cancer, with a projected cost of care to be $174 billion in 2020 [8,10]. Due to the excess life lost and the economic burden of NCDs, it is a national imperative to identify high-risk groups and to institute prevention and early detection strategies among those groups.

Transgender and gender nonbinary (TGNB) are terms used to identify people "whose gender identity and gender role do not conform to what is typically associated with their sex assigned at birth" [11]. TGNB people are part of the broader sexual and gender minority (SGM) community, which also includes lesbian, gay, bisexual, and queer/questioning people. SGM populations are vulnerable to health disparities and inequalities that include higher rates of chronic diseases, worse mental health, and barriers to healthcare when compared with cisgender (not transgender), heterosexual populations [12,13,14,15,16,17,18].

The minority stress theory has been used to help explain how discrimination and stigma experienced by SGM people due to their sexual orientation or gender identity impacts their health [19,20]. The minority stress theory posits that the physical and mental health of SGM people is, at least in part, impacted by recurrent stigma, discrimination, victimization, homophobia, transphobia, and identity concealment [19,20]. Minority stress experienced by TGNB people is evident by a heavier burden of employment discrimination, social stigma and rejection, and violence towards members of the TGNB community. One way that minority stress can be examined is through laws and policies, or lack thereof, available to protect TGNB people from discrimination, stigma, violence, or unequal access to public services [21,22,23]. For example, 23 states in the U.S. do not have laws to prevent employment discrimination based on gender identity, 30 states do not have a law that addresses hate or bias crimes based on gender identity, and 31 states do not have a ban on insurance exclusions for transgender healthcare [24]. Additionally, TGNB people experience stigma and discrimination in many public and social settings, but most concerning for NCD diagnosis and prevention is the excessive discrimination and stigma towards TGNB people in the healthcare setting [25].

Although TGNB people have been identified as a prior group for research [26], there is a lack of health research, especially regarding NCDs, among TGNB populations other than HIV and mental health [27,28]. A systematic review exploring chronic disease or NCD research that has been conducted globally from 1980 through February 2019 found 93 published articles [29]. Eighty percent of the articles examined mental health or substance use/abuse, while 15% studied cardiovascular/cerebrovascular diseases, 12% cancer, 10% respiratory diseases, and 6% chronic liver and kidney disease, illustrating the dramatic lack of research in this area [29]. Moreover, only 6.45% (*n* = 6) of studies used matching of transgender groups with other groups (i.e., cisgender groups) [29]. The lack of NCD research among TGNB people is concerning as they may be at an increased risk for NCDs due to gender-affirming hormone therapy (GAHT) [30], higher rates of health behaviors that are risk factors for chronic diseases (e.g., smoking and heavy alcohol consumption) [30,31], and minority stress. Due to the scarcity of research on NCDs in the TGNB population and lack of matched analyses, the purpose of this study was to use propensity score-matched analysis to assess the burden of chronic conditions among the TGNB population in the U.S.

## 2. Materials and Methods

### 2.1. Dataset

The Behavioral Risk Factor Surveillance Survey (BRFSS) is an annual survey conducted by the Centers for Disease Control and Prevention (CDC) in collaboration with individual U.S. states and territories, including the 50 states, the District of Columbia, Puerto Rico, Guam, American Samoa, Palau, and the U.S. Virgin Islands [32]. It is a random digit dial telephone survey conducted via phone by both landline and cellphone of adults 18 years and older. The CDC is continually working on expanding data collection methods to increase accessibility and representation. The CDC makes publicly available the datasets for research purposes. Each year, the BRFSS includes a series of core questions regarding sociodemographic characteristics, health behaviors, and chronic diseases. In addition to the core questionnaire, the CDC provides 25 optional modules for states and territories to select, including topics to further explore demographics, experiences, and behaviors. States can also include their own questions [32]. The sexual orientation and gender identity (SOGI) module was developed in 2013 by the CDC based on recommendations from the Gender Identity in U.S. Surveillance (GenIUSS) group through the Williams Institute [33], and it was added to the set of optional modules for the 2014 BRFSS survey. To determine gender identity, participants are asked, "Do you consider yourself to be transgender?" If participants responded yes, they were asked a follow-up question to determine their identity from a list of three identifiers: (1) transgender (male-to-female) [transgender women], (2) transgender (female-to-male) [transgender men], or (3) gender nonconforming [nonbinary] [33,34,35].

The BRFSS survey contains sociodemographic characteristics (marital status, education, employment, income, race/ethnicity, age), chronic health condition (asthma, stroke, coronary artery disease, heart attack, skin cancer, other cancer, chronic obstructive pulmonary disease [COPD], depression, arthritis, diabetes, kidney disease), and risk factors for chronic health conditions (blood pressure, cholesterol, alcohol consumption, prediabetes, physical activity, overweight, smoking) [33,34,35]. In 2017, 28 states and territories included the optional SOGI module with 30 states and territories including it in 2018, and 31 states and territories in 2019. From the states that included the SOGI question in 2017, 2018, and 2019, 2827 participants reported they were transgender (by selecting "yes" to the gender identity question), and 661,276 participants reported they were cisgender (by selecting "no" to the gender identity question). The number of cisgender participants was significantly larger than the number of transgender participants, which presents a problem. Significant sample size differences can affect the interpretability and meaningfulness of significance testing by showing significance artificially [36]. Therefore, propensity score matching was employed in the statistical analysis.

### 2.2. Statistical Analysis

Propensity score-matched (PSM) analysis was conducted to minimize selection bias and to account for the characteristics of the two groups being compared [37,38]. The control group (cisgender) and case (transgender) group were matched on a set of variables, including age, race, gender, income, education, and marital status. Propensity matching was conducted through ‘Matchit’ and ‘Tableone’ packages in the R programming software [39]. A logistic regression was fit to estimate propensity scores (predicted probabilities) for each subject [40]. As an intermediary step before matching, the distribution of propensity score across case and control groups was assessed through visual inspection of histogram and jitter plots. Mahalanobis distance was used as a matrix of closeness (distance measure) and optimal caliper width (maximum acceptable distance) was calculated by multiplying standard deviation of the logit-transformed propensity score with 0.2 [37]. Cases and controls were matched using the 1:1 nearest neighbor approach using greedy algorithms. The balance of covariates was assessed through the standardized mean differences (SMD). The SMD values below 0.1 were considered optimal for an adequate covariate balance [37,41,42,43].

Data were assessed for normality assumptions. Continuous data were reported as means and standard deviations (S.D.), while categorical data were reported as frequencies and proportions (%). For outcome analyses, McNemar tests (with continuity correction) were conducted for matched samples [37,41]. A post hoc contingency table analysis using adjusted residuals (or Z scores) was performed in case of multiple comparisons. Bonferroni-corrected p values were generated. Univariate and multivariate logistic regression was conducted to generate unadjusted and adjusted odds ratios. For multivariate logistic regression, variables related to risk factors were used as control variables. The significance level was set at 0.05 level. All analyses were conducted through R and SPSS version 26 software.

## 3. Results

**Figure 1:**
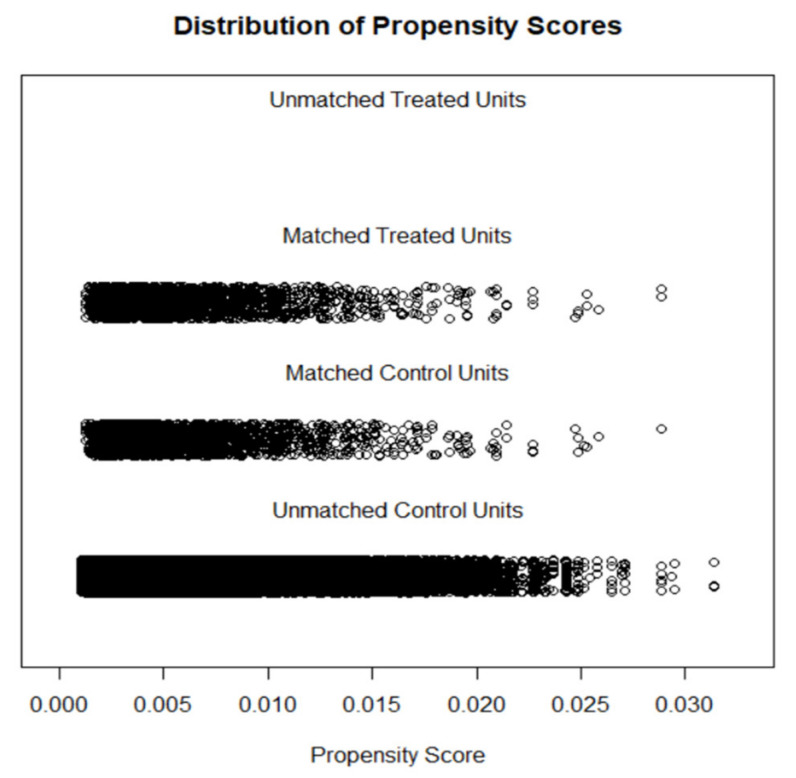
Jitter plot displaying propensity score distribution.

**Figure 2:**
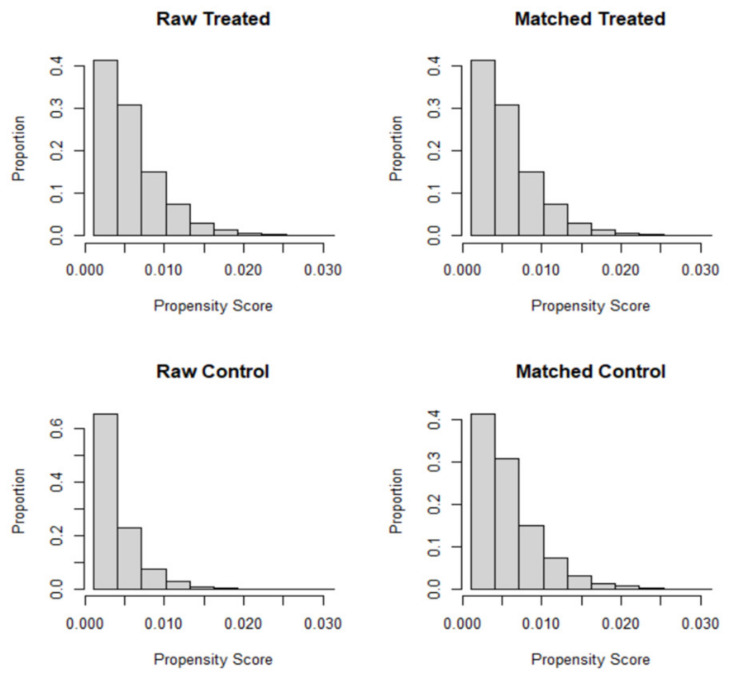
Histograms displaying propensity scores before and after matching.

Among the 664,103 nationally representative weighted sample, 2,827 (0.5%) respondents self-identified as transgender (Table 1). Compared with their cisgender counterparts, the transgender group had lower education (16.0% vs. 27.4%; *p* < 0.001), were younger (27.9% vs. 11.6%; *p* < 0.001), more likely to be African American (14.6% vs. 12.6%; *p* < 0.001) or Hispanic (21.0% vs. 15.8%; *p* < 0.001), had a lower income (24.1% vs. 35.4%; *p* < 0.001), and were more likely to be unemployed (9.0% vs. 5.1%; *p* < 0.001) or unable to work (13.9% vs. 7.2%; *p* < 0.001, Table 1) at baseline. Upon assessing the balancing diagnostics post matching, the balance of covariate distribution was improved with a standardized mean difference lower than 0.1 on all the matching variables (Table 2). Covariate balance and propensity score distribution (pre and post matching) can be visually inspected through Jitter plots and Histogram (Figure 1 and Figure 2). After matching, a total of 2236 transgender people were matched with 2236 cisgender individuals.

In the propensity score-matched sample, significant differences in the prevalence of risk factors were noted (Table 3). Compared with the cisgender group, the transgender group was more likely to report hypertension (31.3% vs. 27.6%; *p* < 0.001), hypercholesteremia (30.8% vs. 23.7%; *p* < 0.001), prediabetes (17.3% vs. 10.3%; *p* < 0.001), and to engaged in heavy drinking (6.7% vs. 6.0%; *p* < 0.001) and smoking (22.4% vs. 20.0%; *p* < 0.001; Table 3). In contrast, the cisgender group was less likely to report physically activity (59.8% vs. 66.3%; *p* < 0.001) and more likely to be overweight (62.0 % vs. 61.7%; *p* < 0.001) than the transgender group (Table 3). The results of outcome analyses indicated that the transgender group was more likely to suffer from chronic health conditions, including asthma (20.1% vs. 19.0%; *p* < 0.001), stroke (6.5% vs. 2.6%; *p* < 0.001), coronary heart disease (7.1% vs. 2.4%; *p* < 0.001), heart attack (7.9% vs. 2.7%; *p* < 0.001), skin cancer (7.0% vs. 3.3%; *p* < 0.001), other types of cancer (8.5% vs. 4.8%; *p* < 0.001), COPD (9.7% vs. 6.8%; *p* < 0.001), diabetes (15.4% vs. 10.7%; *p* < 0.001), kidney diseases (6.1% vs. 3.8%; *p* < 0.001), arthritis (25.5% vs. 17.4%; *p* < 0.001) and depressive disorders (39.4% vs. 19.8%; *p* < 0.001; Table 4).

Odds and adjusted odds ratios comparing transgender and cisgender groups after adjusting for risk factors and health behaviors are shown in Table 5. With cisgender as a reference category, the transgender group was 2.7 times more likely to report depression (aOR: 2.70, 95% CI 2.62–2.72) and more than twice likely to report cardiovascular disorders, including stroke (aOR: 2.52, 95% CI 2.50–2.55), coronary heart disease (aOR: 2.77, 95% CI 2.74–2.81), and heart attack (aOR: 2.90, 95% CI 2.87–2.94). Moreover, the transgender group was more likely to have respiratory disorders, such as asthma (aOR: 1.10, 95% CI 1.10–1.11) and COPD (aOR: 1.50, 95% CI 1.48–1.51). Transgender individuals were 1.5–1.7 times more likely to have diabetes and kidney disorders. Compared with cisgender people, transgender people were nearly twice as likely to suffer from malignant diseases, including skin cancer (aOR: 2.15, 95% CI 2.13–2.18) and other types of cancer (aOR: 1.90, 95% CI 1.88–1.92). 

Table 6 provides the odds and adjusted odds ratios among the un-pooled transgender population after adjusting for risk factors and health behaviors. With cisgender as a reference category, all transgender subgroups (Transgender women, Transgender men, and nonbinary) were 1.4–1.8 times more likely to report asthma. However, transgender men were 32% less likely to have COPD as compared to the cisgender group. Among all transgender subgroups, the nonbinary subgroup was three times more likely to experience coronary heart diseases (aOR: 2.92, 95% CI 2.88–2.97) and heart attack (aOR: 3.28, 95% CI 3.23–3.32) compared with the cisgender group (Table 6). Transgender men were 1.71 times more likely to suffer from depressive disorders than the cisgender group (aOR: 1.71, 95% CI 1.70–1.73). The nonbinary subgroup was 48% less likely to have depression compared with the cisgender group (Table 6). Transgender men were 1.4 times more likely to have kidney diseases (aOR: 1.43, 95% CI 1.41–1.45) compared with the cisgender group. 

## 4. Discussion

This study is among a few to examine multiple chronic diseases of TGNB people compared with cisgender people in a matched sample. Of the six studies that utilized a matched design identified among 93 studies in Rich and colleagues’ 2020 systematic review, only three examined chronic diseases/conditions other than mental health and substance use [29], indicating a knowledge gap. We found a higher rate of all risk factors for chronic diseases, except physical activity and overweight among TGNB people. There were higher rates of all chronic diseases before and after controlling for risk factors among TGNB people than their cisgender peers. However, we found significant differences in subgroup analyses of transgender women, transgender men, and nonbinary groups compared with the cisgender group.

Adjusted odds revealed that the TGNB group and all transgender subgroups were more likely to report coronary heart disease and heart attack than the cisgender group after controlling for risk factors for coronary health disease and heart attack, including smoking, high blood pressure, and high cholesterol. This confirms findings from other studies that used data from large, randomly sampled, nationally representative datasets [29,44,45]. There may be an association between coronary heart disease and GAHT; however, the evidence is equivocal. More long-term follow-up with older groups of transgender people on GAHT is needed [30]. Transgender men often take testosterone which has been shown to increase risk for heart disease, especially among older men [30]. Although we found higher rates of stroke among transgender men in this study, other research regarding GAHT and stroke among transgender people shows a slight increased risk for transgender women and not transgender men [46]. Findings are mixed regarding testosterone therapy and stroke [47]; however, there does seem to be an increased risk of stroke associated with long-term estrogen use [46] and more research is needed concerning GAHT and stroke. For transgender women, estrogen-progestin is not suggested due to an increased risk of heart attack and stroke among cisgender women [30].

Again, TGNB people are more likely to experience minority stress (stigma, discrimination, victimization, homophobia, transphobia, and identity concealment) [19,20]. Flentje and colleagues have proposed a conceptual model of how minority stress may impact biological functions and clinical outcomes [48]. Exposures to discriminating and stigmatizing events may increase the allostatic load and lead to cardiovascular dysfunction. Indeed, one study showed that exposure to moderate to high levels of minority stress resulted in differential expressions of genes and pathways related to cardiovascular function and cancer [49]. More research is needed to identify the causal pathway between minority stress and chronic diseases such as heart disease. 

All transgender subgroups were more likely to report asthma compared with the cisgender group. Other studies have also found a higher rate of asthma among transgender populations, especially among those on GAHT [50,51]. Although the hypothesis that transgender men would have a lower risk of asthma and transgender women would have a higher risk due to the pathogenic role of estrogens and the protective role of androgens among cisgender people, Morales-Estrella and colleagues found an increased risk of asthma among both transgender men and women on GAHT in their study [51]. They concluded that there might be a respiratory health risk associated with GAHT. Data were not available about GAHT in this dataset, and more research is needed to identify the unique risk of asthma for transgender people who are on GAHT and those who are not GAHT.

As a group, transgender people were more likely to report both skin cancer and other cancers. However, subgroup analyses found that only transgender men had an increased risk for skin cancer, with transgender women and nonbinary groups having lower risks than the cisgender group. Because of the strong association between sun exposure and skin cancer [52], these results may point to greater exposure to the sun for transgender men earlier in their life or greater sensitivity to the sun. Both transgender men and women had an increased risk for other cancers. Other research has found higher rates of some cancers among transgender women and men than cisgender women and men. Specifically, transgender women have a higher risk of endocrine and viral infection-induced cancers than cisgender men and higher risk of lymphatic and hematopoietic cancers than cisgender women [53]. Transgender men have a higher risk of breast cancers, smoking, and viral infection-related (cervical) cancers than cisgender men [53]. 

While the mechanism for differences in some of these cancers is not fully known, excessive exposure to minority stress may increase cancer risk through alterations in biological functions such as inflammation and the immune function [48]. Additionally, more research on GAHT as a potential link to cancer among transgender men and women is needed as most research to this point has included relatively young cohorts [54] who were on GAHT for short durations, and findings are inconclusive. However, our findings do highlight the importance of providing TGNB people age-specific cancer screenings. Unfortunately, TGNB people experience disproportionate discrimination and stigma in healthcare settings which includes a lack of health insurance, being denied services, and even physical or verbal abuse from providers [25], resulting in barriers to primary care and preventive services. Policies and practices within the healthcare setting need to be re-examined and modified to provide inclusive care for TGNB people by removing barriers to care. For example, barriers to cervical cancer screenings for transgender men need to be mitigated as they experience disparities in cervical cancer screening compared with cisgender women, although the risk of cervical cancer is comparable [55].

Transgender men and women were at a higher risk for arthritis, kidney disease, and depression, while transgender women were at higher risk for diabetes and COPD when compared with the cisgender group. Disparities in these chronic diseases between transgender and cisgender populations are consistent with other research [56]. There is a lack of research on the cause of these differences, and more research is needed to understand the increased risk for many chronic diseases among TGNB people, particularly by subgroups. However, these findings point to the need for access to healthcare and disease prevention for transgender people. Unfortunately, TGNB people face discrimination and even hostility within the healthcare system [57], and are therefore more likely to delay or forgo healthcare. This discrimination may occur because they do not adhere to the normative gender expectations based on their assigned sex at birth. Research has found that transgender people who have a non-inclusive primary care physician or who delay medical care due to fear of discrimination were less likely to have had a medical check-up in the past two years [50]. Additionally, over one-quarter of transgender people have been denied access to healthcare by a provider and one-third have had to educate their provider about transgender care. Transgender patients are more likely to experience both verbal and physical abuse at the hands of their healthcare providers than other groups of patients [25]. While there is limited research about the phenomenon of poorer medical care provided to TGNB people by the medical community, some posit that the medical community reflects the transphobic, cisgenderist, and heterosexist attitudes and behaviors found in society. Although there has been a "call to action" to include culturally competent care for transgender and nonbinary patients in medical education curriculum [58], there are still gaps in this training for all health professionals, both those currently in school and those who are practicing. There is a need for healthcare providers and medical systems to see past the binary categories of gender and to create an inclusive environment for TGNB patients. Additionally, little is known about healthcare providers’ prospective of and barriers to carrying for TGNB patients, and work needs to be done in this area.

### Strengths and Limitations

Of the limited number of studies examining chronic conditions (other than mental health and substance use) among transgender adults compared with cisgender adults, most have used unmatched data [29], resulting in a significant sample size difference between groups. This could generate biased estimates [36]. A strength of this study was the use of a national sample weighted to represent the U.S. adult population. Additionally, transgender and cisgender participants were matched using PSM on a 1:1 ratio. This helped to minimize selection bias and controlled for differences in demographic characteristics of two groups (transgender and cisgender). Despite these strengths, there are limitations to the current study. This was a cross-sectional design; therefore, causation cannot be inferred. Data were self-reported and subject to self-report bias. Additional limitations include social desirability bias and recall bias. Participants may have failed to report or incorrectly reported chronic diseases, risk factors, demographic characteristics, or gender identity either intentionally or unintentionally. The BRFSS does not include data about gender-affirming surgical history (e.g., gonadectomy, total hysterectomy, mastectomy), which limits our ability to draw conclusions about comparative risks. Lastly, the transgender and cisgender larger groups were matched by demographic characteristics; however, there may have been differences in these characteristics in the subgroups, resulting in differences between subgroups.

## 5. Conclusions

This study highlights the disparities in NCDs and risk factors for NCD among TGNB adults compared with cisgender adults in the U.S. This is concerning because TGNB people are less likely to seek out medical care due to fear of discrimination or harassment, a lack of health insurance, or cost of care. To reduce premature mortality and the economic burden associated with NCDs, both in the U.S. and globally, increased effort is needed to make healthcare and disease prevention programs accessible and comfortable for people who identify as TGNB through culturally competent care for TGNB patients. The U.S. took a step in the right direction in May 2021 as the Biden administration reinstated protection from discrimination in healthcare based on sexual orientation or gender identity through section 1557 of the Affordable Care Act. However, discriminatory laws and policies have been proposed in several states which include preventing pediatricians from providing transgender care to their patients. Transphobic laws only increase the stigmatization and discrimination of transgender people adding to the minority stress that they experience over the life course. Laws and policies are needed that protect rather than harm TGNB people and provide them with the needed access to preventive care to address the unequal burden of NCDs. Research is needed to understand why TGNB people are provided suboptimal care by healthcare providers and within the healthcare system. While GAHT may increase the risk of some chronic diseases for transgender people, further exploration is needed to determine whether there is an association. Additionally, more research is needed to understand unique risk factors for chronic diseases among TGNB people beyond known risk factors (e.g., smoking, heavy alcohol consumption, physical inactivity). These findings offer an evidence base to build health equity models to reduce disparities among transgender groups. 

## Figures and Tables

**Table 1:** Baseline characteristics of the sample population.

Characteristics	Gender Identities		Total	*p* Value *
	Transgender	Cisgender		
	*n* (Weighted %)	*n* (Weighted %)	*n* (Weighted %)	
All	2827 (0.5)	661,276 (99.5)	664,103	
**Birth-Assigned Sex**				
Male	1456 (51.9)	294,479 (47.7)	295,935 (48.0)	<0.001
Female	1324 (48.1)	366,243 (51.7)	367,567(52.0)	
**Sexual Orientation**				
Straight	1613 (51.9)	615,018 (94.5)	616,631 (94.3)	<0.001
Lesbian or gay	272 (10.8)	10,462 (1.8)	10,734 (1.8)	<0.001
Bisexual	445 (21.9)	13,269 (2.7)	13,714 (2.8)	<0.001
Other	371 (15.4)	5743 (1.0)	6114 (1.1)	<0.001
**Education**				
Did not graduate high school	412 (22.8)	46,504 (13.1)	46,916 (13.2)	<0.001
High school graduate	931 (32.3)	177,238 (28.3)	178,169 (28.3)	<0.001
Attended college	723 (28.9)	183,259 (31.3)	183,982 (31.3)	<0.001
College graduate	748 (16.0)	252,304 (27.4)	253,052 (27.3)	<0.001
**Age (in years)**				
18–24	426 (27.9)	35,274 (11.6)	35,700 (11.7)	<0.001
25–34	421(19.5)	63,735(16.0)	64,156 (16.0)	
35–44	328 (12.1)	74,941(16.0)	75,269 (15.9)	<0.001
45–54	372 (13.0)	97,425 (16.3)	97,797 (16.3)	<0.001
55–64	509 (12.6)	139,577 (17.7)	140,086(17.7)	<0.001
65–74	427 (8.2)	139,471 (13.2)	139,898 (13.2)	<0.001
75 or above	307 (6.7)	101,102 (9.2)	101,409 (9.2)	<0.001
**Race/Ethnicity**				
White/Caucasian	1784 (55.8)	500,935 (63.7)	502,719 (63.7)	<0.001
Black/African American	315 (14.6)	56,220 (12.6)	56,535 (12.6)	<0.001
Hispanic	337 (21.0)	46,450 (15.8)	46,787 (15.9)	<0.001
Other including multiracial, Asian, NH/PI, AI/AN	365 (8.6)	53,387 (7.9)	53,752 (7.9)	<0.001
**Income**				
<10 K	214 (12.0)	23,795 (5.1)	24,009 (5.1)	<0.001
10–25 K	712 (31.7)	115,054 (21.2)	115,766 (21.3)	<0.001
25–50 K	573 (19.7)	134,271 (23.2)	134,844 (23.2)	<0.001
50–75 K	311 (12.5)	89,208 (15.1)	89,519 (15.0)	<0.001
>75 K	515 (24.1)	192,787 (35.4)	193,302 (35.3)	<0.001
**Employment**				
Employed	1350 (49.2)	326,136 (56.8)	327,486 (56.8)	<0.001
Unemployed	203 (9.0)	26,513 (5.1)	26,716 (5.2)	<0.001
Out of labor force (e.g., retired, homemakers, and students)	888 (27.9)	254,395 (30.8)	255,283 (30.8)	<0.001
Unable to work	353 (13.9)	48,893 (7.2)	49,246 (7.3)	<0.001

* *p* values are Bonferroni corrected.

**Table 2:** Comparing the covariates’ balance diagnostics (effectiveness of propensity score matching).

Unmatched				Matched		
	Transgender	Cisgender	SMD	Transgender	Cisgender	SMD
*n*	2827	661,276		2236	2236	
Age	2.94 (1.97)	3.63 (1.75)	0.372	3.01 (1.91)	3.02 (1.91)	0.004
Gender	1.48 (0.50)	1.55 (0.50)	0.157	1.46 (0.50)	1.46 (0.50)	<0.001
Race	1.85 (1.35)	1.53 (1.12)	0.260	1.83 (1.34)	1.83 (1.34)	<0.001
Marital status	2.96 (1.85)	2.28 (1.63)	0.390	2.85 (1.84)	2.85 (1.84)	0.001
Education level	2.64 (1.03)	2.97 (0.97)	0.332	2.67 (1.03)	2.67 (1.03)	<0.001
Income	3.09 (1.30)	3.56 (1.27)	0.370	3.11 (1.29)	3.11 (1.30)	<0.001
Employment	2.09 (1.14)	2.04 (1.09)	0.043	2.05 (1.14)	2.05 (1.14)	0.001

**Table 3:** Prevalence of risk factors in matched cohorts.

Matched			
Outcome	Transgender *n* (Weighted %)	Cisgender *n* (Weighted %)	*p* Value *
**High blood pressure**			
Yes	544 (31.3)	848 (27.6)	<0.001
No	834 (68.7)	1283 (72.4)	
**High cholesterol**			
Yes	434 (30.8)	651 (23.7)	<0.001
No	802 (69.2)	1281(76.3)	
**Heavy alcohol consumption**			
Yes	114 (6.7)	164 (6.0)	<0.001
No	1238 (93.3)	1932 (94.0)	
**Prediabetes**			
Yes	146 (17.3)	51 (10.3)	<0.001
No	944 (82.7)	303 (89.7)	
**Physical activity**			
Yes	1539 (66.3)	1605 (59.8)	<0.001
No	675 (33.7)	597 (40.2)	
**Overweight**			
Yes	1412 (61.7)	1413 (62.0)	<0.001
No	689 (38.3)	707 (38.0)	
**Current smoker**			
Yes	450 (22.4)	402 (20.0)	<0.001
No	1751(77.6)	1803 (80.0)	

* McNemar test.

**Table 4:** Outcome analysis in matched cohorts.

Matched			
Outcome	Transgender *n* (Weighted %)	Cisgender *n* (Weighted %)	*p* Value *
**Asthma (Current)**			
Yes	406 (20.1)	412 (19.0)	<0.001
No	1822 (79.9)	1817 (81.0)	
**Stroke**			
Yes	133 (6.5)	98 (2.6)	<0.001
No	2090 (93.5)	2133 (97.4)	
**Coronary Heart disease**			
Yes	153 (7.1)	107 (2.4)	<0.001
No	2056 (92.9)	2108 (97.6)	
**Heart Attack**			
Yes	175 (7.9)	126 (2.7)	<0.001
No	2044 (92.1)	2097 (97.3)	
**Cancer (Skin Cancer)**			
Yes	176 (7.0)	154 (3.3)	<0.001
No	2048 (93.0)	2076 (96.7)	
**Cancer (Other)**			
Yes	187 (8.5)	195 (4.8)	<0.001
No	2038 (91.5)	2035 (95.2)	
**COPD**			
Yes	234 (9.7)	221 (6.8)	<0.001
No	1995 (90.3)	2004 (93.2)	
**Depression**			
Yes	756 (39.4)	455 (19.8)	<0.001
No	1465 (60.6)	1770 (80.2)	
**Arthritis**			
Yes	714 (25.5)	666 (17.4)	<0.001
No	1513 (74.5)	1561 (82.6)	
**Diabetes**			
Yes	375 (15.4)	328 (10.7)	<0.001
No	1824 (84.6)	1878 (89.3)	
**Kidney Diseases**			
Yes	120 (6.1)	99 (3.8)	<0.001
No	2105 (93.9)	2131 (96.2)	

* McNemar Test.

**Table 5:** Adjusted and unadjusted odds ratio for chronic health conditions among matched samples.

Variable	Unadjusted Odds Ratio	95% CI		*p* Value	AOR	95% CI		*p* Value
		LCL	UCL			LCL	UCL	
**Asthma (Current)**								
Transgender	1.10	1.06	1.11	<0.001	1.10	1.10	1.11	<0.001
Cisgender	REF	-	-	-	-	-	-	-
**Stroke**								
Transgender	2.65	2.61	2.70	<0.001	2.52	2.50	2.55	<0.001
Cisgender	REF	-	-	-	-	-	-	-
**Coronary Heart Disease**								
Transgender	3.12	3.08	3.16	<0.001	2.77	2.74	2.81	<0.001
Cisgender	REF	-	-	-	-	-	-	-
**Heart attack**								
Transgender	3.08	3.04	3.11	<0.001	2.90	2.87	2.94	<0.001
Cisgender	REF	-	-	-	-	-	-	-
**Skin Cancer**								
Transgender	2.17	2.15	2.20	<0.001	2.15	2.13	2.18	<0.001
Cisgender	REF							
**Other Cancers**								
Transgender	1.90	1.83	1.91‘	<0.001	1.90	1.88	1.92	<0.001
Cisgender	REF	-	-	-	-	-	-	-
**COPD**								
Transgender	1.50	1.46	1.51	<0.001	1.50	1.48	1.51	<0.001
Cisgender	REF	-	-	-	-	-	-	-
**Depression**								
Trans	2.62	2.61	2.64	<0.001	2.70	2.62	2.72	<0.001
Cis	REF							
**Arthritis**								
Transgender	1.62	1.62	1.63	<0.001	1.70	1.68	1.70	<0.001
Cisgender	REF	-	-	-	-	-	-	-
**Diabetes**								
Transgender	1.52	1.51	1.53	<0.001	1.50	1.49	1.51	<0.001
Cisgender	REF	-	-	-	-	-	-	-
**Kidney Disease**								
Transgender	1.70	1.64	1.71	<0.001	1.70	1.67	1.71	<0.001
Cisgender	REF	-	-	-	-	-	-	-

*p* values less than 0.05 are statistically significant; adjusted odds ratios (AOR) were obtained after controlling for risk factors; LCL—Lower Confidence Limit; UCL—Upper Confidence Limit.

**Table 6:** Adjusted and unadjusted odds ratio for chronic health conditions among matched sample of un-pooled transgender population.

Variable	Unadjusted OR	95% CI (LCL, UCL)	*p* Value	AOR	95% CI (LCL, UCL)	*p* Value
**Asthma (Current)**						
Transgender Women	1.60	1.59, 1.61	<0.001	1.81	1.80, 1.83	<0.001
Transgender Men	1.59	1.57, 1.60	<0.001	1.50	1.48, 1.51	<0.001
Nonbinary	1.25	1.24, 1.25	<0.001	1.41	1.40, 1.41	<0.001
Cisgender	REF	-	-	-	-	-
**Stroke**						
Transgender Women	0.73	0.72, 0.74	<0.001	0.95	0.93, 0.96	<0.001
Transgender Men	1.23	1.21,1.25	<0.001	1.33	1.31, 1.35	<0.001
Nonbinary	0.37	0.36,0.38	<0.001	0.48	0.47, 0.49	<0.001
Cisgender	REF	-	-	-	-	-
**Coronary Heart Disease**						
Transgender Women	1.71	1.69, 1.74	<0.001	1.20	1.18, 1.22	<0.001
Transgender Men	1.22	1.20, 1.24	<0.001	1.09	1.02, 1.19	<0.001
Nonbinary	3.85	3.80,3.91	<0.001	2.92	2.88, 2.97	<0.001
Cisgender	REF	-	-	-	-	
**Heart Attack**						
Transgender Women	1.99	1.96, 2.02	<0.001	1.51	1.49, 1.53	<0.001
Transgender Men	1.09	1.08, 1.11	<0.001	1.03	1.02, 1.05	<0.001
Nonbinary	3.84	3.79,3.89	<0.001	3.28	3.23, 3.32	<0.001
Cisgender	REF	-	-	-	-	-
**Skin Cancer**						
Transgender Women	0.55	0.54, 0.56	<0.001	0.62	0.61, 0.63	<0.001
Transgender Men	1.02	1.01,1.04	0.002	1.04	1.03, 1.06	<0.001
Nonbinary	0.39	0.38, 0.39	<0.001	0.47	0.46, 0.47	<0.001
Cisgender	REF	-	-	-	-	-
**Other Cancers**						
Transgender Women	0.89	0.88,0.90	<0.001	1.08	1.07, 1.10	<0.001
Transgender Men	1.11	1.10, 1.12	<0.001	1.10	1.07, 1.11	<0.001
Nonbinary	0.53	0.53,0.54	<0.001	0.68	0.67, 0.69	<0.001
Cisgender	REF	-	-	-	-	-
**Chronic Obstructive Pulmonary Disease**						
Transgender Women	0.89	0.88,0.90	<0.001	1.17	1.15, 1.18	<0.001
Transgender Men	0.66	0.65,0.67	<0.001	0.68	0.67, 0.69	<0.001
Nonbinary	0.59	0.59,0.60	<0.001	0.78	0.77, 0.79	<0.001
Cisgender	REF	-	-	-	-	-
**Depression**						
Transgender Women	1.39	1.38,1.40	<0.001	1.43	1.42, 1.44	<0.001
Transgender Men	1.73	1.72,1.74	<0.001	1.71	1.70, 1.73	<0.001
Nonbinary	0.50	0.49,0.50	<0.001	0.52	0.51, 0.52	<0.001
Cisgender	REF	-	-	-	-	-
**Arthritis**						
Transgender Women	1.08	1.07, 1.09	<0.001	1.31	1.30, 1.32	<0.001
Transgender Men	1.08	1.07, 1.09	<0.001	1.11	1.10, 1.12	<0.001
Nonbinary	0.66	0.64, 0.67	<0.001	0.78	0.78, 0.79	<0.001
Cisgender	REF	-	-	-	-	-
**Diabetes**						
Transgender Women	0.83	0.82,0.83	<0.001	1.18	1.17, 1.19	<0.001
Transgender Men	0.56	0.55,0.56	<0.001	0.62	0.61, 0.62	<0.001
Nonbinary	0.55	0.55,0.57	<0.001	0.65	0.64, 0.66	<0.001
Cisgender	REF	-	-	-	-	-
**Kidney Disease**						
Transgender Women	0.95	0.94,0.96	<0.001	1.18	1.16, 1.20	<0.001
Transgender Men	1.40	1.37,1.42	<0.001	1.43	1.41, 1.45	<0.001
Nonbinary	0.65	0.64,0.66	<0.001	0.84	0.83, 0.85	<0.001
Cisgender	REF	-	-	-	-	-

*p* values less than 0.05 are statistically significant; adjusted odds ratios (AOR) were obtained after controlling for risk factors; LCL—Lower Confidence Limit; UCL—Upper Confidence Limit.

## Data Availability

Data are available in a publicly accessible repository that does not issue DOIs Publicly available datasets were analyzed in this study. These data can be found here: https://www.cdc.gov/brfss/data_documentation/index.htm (accessed on 6 April 2021).
